# Sleep-related breathing disorder in non-infectious pulmonary complications after pediatric allogeneic stem cell transplantation

**DOI:** 10.1038/s41390-022-02339-7

**Published:** 2022-10-25

**Authors:** Elli-Maija Ukonmaanaho, Turkka Kirjavainen, Laura Martelius, Jouko Lohi, Riitta Karikoski, Minna Koskenvuo, Mervi Taskinen

**Affiliations:** 1 Division of Pediatric Hematology and Oncology, Oulu University Hospital, Oulu, Finland; 2 University of Helsinki, Helsinki, Finland; 3 Division of Pediatric Pulmonology, New Children’s Hospital, University of Helsinki and Helsinki University Hospital, Helsinki, Finland; 4 Division of Radiology, New Children’s Hospital, University of Helsinki and Helsinki University Hospital, Helsinki, Finland; 5 Division of Pathology, University of Helsinki and Helsinki University Hospital, Helsinki, Finland; 6 Division of Pathology, Helsinki University Hospital, Helsinki, Finland; 7 Division of Hematology-Oncology and Stem Cell Transplantation, New Children’s Hospital, University of Helsinki and Helsinki University Hospital, Helsinki, Finland

## Abstract

**Background:**

Chronic lung problems are a rare but serious complication of allogeneic hematopoietic stem cell transplantation (HSCT). We studied clinical phenotypes and polysomnography appearance of breathing abnormality in late onset non-infectious pulmonary complications (NIPS).

**Methods:**

We reviewed Finnish national reference database between the years 1999 and 2016. We identified 12 children with most severely decreased pulmonary function and performed polysomnography and 24 aged-matched controls out of 325 performed pediatric allogeneic HSCTs.

**Results:**

All patients with NIPS had severely decreased pulmonary function already at 6 months post HSCT with median FEV_1_ value 42% (interquartile range (IQR) 30–52%) of predicted normal values. Seven children had obstructive and five children more restrictive lung function. Children with obstructive lung function showed laborious breathing (7/7), decreased oxygenation and ventilation-to-perfusion mismatch (6/7), or REM-sleep-related hypoventilation (4/7) on polysomnography. Children with restrictive lung function (5/12) did not show sleep-related breathing disorder.

**Conclusions:**

Children going through allogeneic HSCT who develop severe chronic obstructive lung function are more likely to present with sleep-related hypoxia and hypoventilation than children with restrictive lung function.

**Impact:**

- Children with severe obstructive lung function and chronic lung graft-versus-host disease following hematopoietic stem cell transplantation are more likely to present with sleep-related mild hypoxia and hypoventilation than children with restrictive lung disease.
- To our knowledge there are no reports on sleep-related breathing disorders and ventilatory function measured by polysomnography in children with pulmonary complications after allogeneic HSCT.
- Polysomnography may add to the differential diagnostics between patients with BOS and other non-infectious pulmonary complications.

## Introduction

Allogeneic hematopoietic stem cell transplantation (HSCT) gives a widely accepted curative option for severe hematological malignancies and non-malignant diseases.^1^ The outcome of HSCT has improved along with the development of preparative regimens, advances in histocompatibility matching, improved prophylaxis, treatment of graft-versus-host disease (GvHD), and improved control of infections. Over the years, the preparative regimen has shifted from myeloablative conditioning to reduced intensity regimens to have a better control over the wide spectrum of acute and long-term toxicities.^1^ Still, toxic treatment options such as total body irradiation (TBI) remains in the mainstay regimens for pediatric acute lymphoblastic leukemia.^2^

Pulmonary complications following allogeneic HSCT are divided into infectious and non-infectious complications.^3,4^ Late-onset non-infectious pulmonary complications (NIPS) include obliterative bronchiolitis syndrome (BOS), cryptogenic organizing pneumonia (COP), and idiopathic pneumonia syndrome (IPS).^4,5^ BOS is a rare but still the most frequent severe long-term lung complication after HSCT.^6–8^ In general, it holds a poor prognosis.^6–8^ BOS is an obstructive process causing inflammation and fibrosis to the bronchial walls leading to small bronchiolar intraluminal obstruction.^9,10^ The risk factors for BOS include acute and chronic GvHD, mismatched human leukocyte antigen (HLA) donor, abnormal pre-HSCT pulmonary function, GvHD prophylaxis with methotrexate, conditioning with busulfan, low immunoglobulin level (IgG < 7 g/L) post-HSCT, and respiratory viral infections within the first 100 days following transplant.^4,9,11^ BOS is the entity formally recognized as a manifestation of lung chronic GvHD (cGvHD).^12^

Lung function of patients diagnosed with BOS is poor, but little attention has been paid to its consequences.^4,7,8^ Hypoventilation during REM sleep in pediatric patients is common for example in neuromuscular diseases, where nighttime hypoventilation is reported at lung function levels less than FVC 50% predicted values.^13–15^

To our knowledge, there are no reports on sleep-related breathing disorders and ventilatory function measured by polysomnography in children with pulmonary complications after allogeneic HSCT.

The purpose of our study is to characterize the most severe long-term pediatric non-infectious lung complications after allogeneic HSCT with focus on pulmonary function, imaging, histological characteristics and ventilatory function measured by polysomnography.

## Materials and methods

**Fig. 1:**
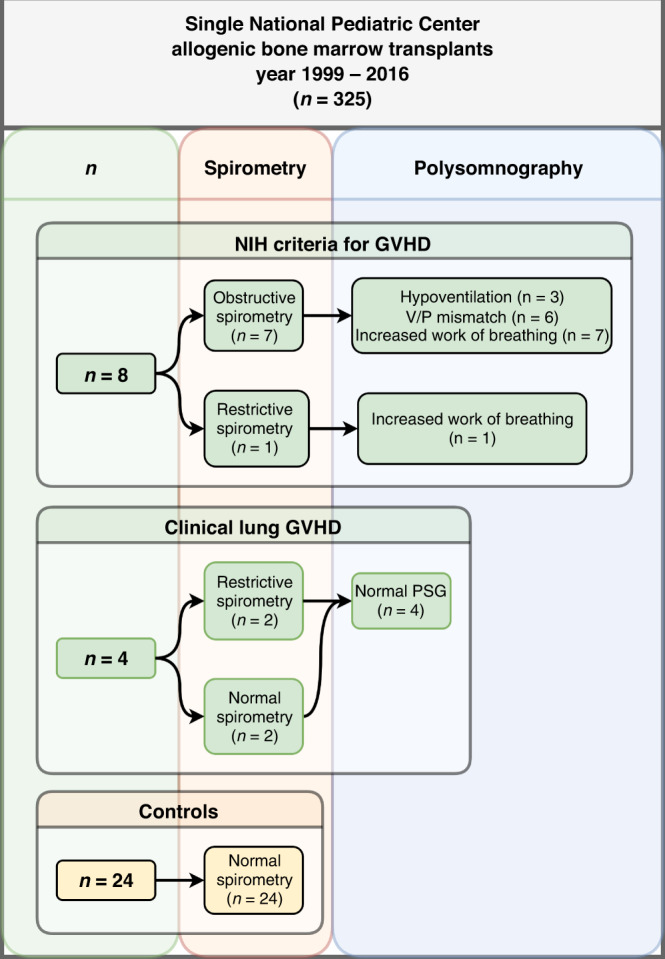
Study flow chart. NIH The National Institution of Health, GvHD graft-versus-host disease, V/P ventilation to perfusion, PSG polysomnography.

This retrospective study was performed at Hospital for Children and Adolescents, Helsinki University Hospital, Finland. Altogether, 325 pediatric allogeneic HSCTs were performed during study period from 1999 to 2016. We re-analyzed pulmonary function testing (PFT), high-resolution lung computer tomography images (HRCT), and polysomnography recordings performed on clinical basis to the pediatric allogeneic HSCT patients with severely decreased lung function. We identified 13 children with long-term severe pulmonary complications who had undergone polysomnography evaluation. One patient was excluded from this analysis since polysomnography had been performed in another sleep laboratory. For each studied children, we searched two aged-, underlying disease-, and preparative regimen-matched control patients transplanted in our center, but who had no lung symptoms. Polysomnography was not done to the controls. Study flow chart is presented in Fig. 1.

### Clinical data

The clinical data collected from the local HSCT database and from the patient charts included patient demographic and underlying disease characteristics, HSCT characteristics with information about conditioning regimen and all other medication used, donor and graft characteristics, engraftment, and acute and chronic GvHD data. We collected data of all respiratory symptoms and infectious complications after HSCT prior to the onset of the lung symptoms. Lung symptoms registered were cough, shortness of breath and difficulty of breathing without any infectious agent present. All patients had sulfamethoxazole-trimethoprim prophylaxis against *Pneumocystis jirovecii* (Table 1).

**Table 1:** Demographic data of the studied children and controls.

Characteristic	Case group *N* = 12	Control group *N* = 24	*p* value
Relapsed disease	1 (8%)	5 (21%)	0.32
Median age at HSCT (IQR)	8.4 (6.4–11.5)	7.5 (5.2–10.3)	0.50
Sex (%)			
Female	4 (33%)	12 (50%)	0.34
Male	8 (67%)	12 (50%)	
Pretreatment (%)			
Cyclophosphamide based	6 (50%)	11 (46%)	0.80
Cytarabine based	5 (42%)	8 (33%)	
Other	1 (8%)	5 (21%)	
TBI (%)			
10 Gy	9 (75%)	20 (83%)	0.66
>10 Gy	3 (25%)	4 (17%)	
Lung shielding during TBI (%)	6 (50%)	11 (46%)	0.82
Donor (%)			
Matched URD	7 (58%)	14 (58%)	1.00
Matched family	5 (42%)	10 (42%)	
GvHD prophylaxis (%)			
Cyclosporine based	9 (75%)	23 (96%)	0.98
Other	3 (25%)	1 (4%)	
Viral infections			
CMV	7 (58%)	2 (8%)	0.29
Adeno	2 (17%)	1 (4%)	
Resp	4 (33%)	0 (0%)	
Acute graft-versus-host-disease (%)			
Grade 0–1	3 (25%)	18 (75%)	0.004
Grade 2–4	9 (75%)	6 (25%)	
Chronic GvHD other than lungs	11 (92%)	7 (29%)	<0.001
Exitus	3 (25%)	3 (13%)	0.37

*HSCT* hematopoietic stem cell transplantation, *TBI* total body irradiation, *URD* unrelated donor, *CMV* cytomegalovirus, *Resp* respiratory viruses in the nasopharyngeal secrete, *GvHD* graft-versus-host disease.

TBI was fractionated in 2 Gy fractions, two fraction per day. Patients at or over 10 years had lung shielding capping the lung irradiation dose at 8 Gy.

### Pulmonary function

The follow-up schema for pulmonary function performed by spirometry was before HSCT, at 6 months, 1 year, and 2–5 years after the transplantation. We included measurements of forced vital capacity (FVC), forced expiratory volume in 1 s (FEV_1_), forced expiratory flow 50% (FEF_50_), peak expiratory flow (PEF), and calculated the relative change between pre- and post-HSCT. Single breath diffusion capacity measurement was included in children with reliably performed spirometry and FVC over 1 liter. Diffusion capacity parameters of total lung capacity (TLC), residual volume (RV), and corrected diffusion capacity (DLCOc) were included.

Lung HRCT was taken if BOS was suspected. Radiologist (L.M) analyzed HRCT images blinded to the clinical history. The presence of mosaic pattern (MMO), bronchial wall thickening (BWT), bronchial dilation (BD), ground glass opacity (GGO), and for the study purposes air-trapping were recorded in six different lung areas: giving a full score ranging from 0 (no lobe affected) to 6 (all lobes affected). Lung biopsies were analyzed by two pediatric pathologists blinded to the clinical history (J.L., R.K.).

### Polysomnographies

Polysomnography (PSG) was performed in pediatric sleep laboratory to patients with chronic lung symptoms and severe deterioration in PFT follow-up. The PSG comprised monitoring of two to four electroencephalography (EEG) channels (Cz-Fz, Cz-O2, C4-M1, O2-M1), two electrooculography (EOG) channels (right and left), chin and diaphragm electromyography (EMG), nasal airflow (pressure transducer), respiratory movements (thoracic and abdominal bands), electrocardiography (ECG), pulse oximeter oxyhemoglobin saturation (SpO_2_) by one or two pulse oximeters with 2–4 s averaging interval (Embla and Masimo Radical Pulse CO-Oximeter, Masimo, Irvine, CA), end-tidal carbon dioxide (EtCO_2_) (CAP10 Capnograph, Medlab medizinische Diagnosegeräte GmbH, Germany), position sensor, and synchronized video recordings. Some recordings included also transcutaneous carbon dioxide (TcCO_2_) (SenTec, St.Louis, MO).

All PSG recordings were reanalyzed and scored by an experienced scorer (T.K.) using Embla® RemLogic™ (Natus Medical Inc., Pleasanton, CA). The sleep stage analysis was performed visually, applying the AASM scoring stage criteria (22) {Berry, 2012 #132}.

### Diagnosis of BOS

We analyzed the children with NIPS by using the original and modified National Institution of Health (NIH) criteria.^9,16^ The original NIH criteria required demonstration of the characteristic findings of airflow obstruction by: (1) FEV_1_ <75% of predicted value together with an irreversible ≥10% decline in FEV_1_ in <2 years, (2) evidence of airway obstruction with FEV_1_/FVC ratio <0.7 or the lower limit of the 90% confidence interval of the ratio, (3) absence of infection, and (4) (a) another manifestation of chronic GvHD, (b) air trapping on expiratory CT, or (c) residual volume (RV) over 120% or RV/total lung capacity (TLC) ratio exceeding the 90% confidence interval limit.^16^ The original NIH criteria for BOS have been re-evaluated by Chien et al., and the modified criteria take into account that BOS patients may have falsely elevated FEV_1_/FVC ratio due to dynamic airway obstruction (early collapse of airways during forced expiration).^9^

### Statistical analysis

Statistical analyses were made by using IBM SPSS Statistics, version 25 (Armonk, NY). Nonparametric tests were used. The demographic data were compared between groups by using Fisher’s exact test, Chi-square-test, and two-sample *z-*test to compare sample proportion. Between-group analyses were done with Mann–Whitney *U*-test. Repeated test results were tested using paired sample Wilcoxon Signed Rank Test. *p* values of 0.05 or below were considered significant.

## Results

**Fig. 2:**
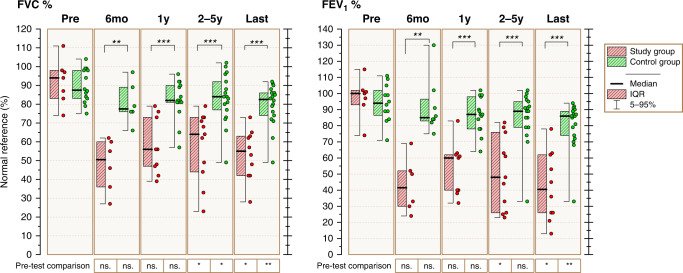
Lung function before and after allogeneic hematopoietic stem cell transplantation. Statistical significance of comparisons between the two groups is marked on top of the figure, and significance of within-group comparisons below the figure. FVC functional vital capacity, FEV_1_ forced expiratory volume at 1 s, ns non-significant, *p < 0.05, **p < 0.01, ***p < 0.001.

All 12 patients with NIPS had severely decreased pulmonary function already at 6 months post-HSCT with median FEV_1_ value 42% of predicted normal value (IQR 30–52). Median pre-HSCT FEV_1_ in children with NIPS was 100% of predicted normal mean [interquartile range (IQR) 93–102%)] and controls FEV_1_ 94% (IQR 87–102%) (*p* = 0.54). Median FVC and FEV_1_ values were significantly lower in children with NIPS than in controls at all post-HSCT follow-up time points (Fig. 2).

All patients had hematological malignancy as underlying disease. There were no statistically significant differences in conditioning chemotherapy, use of total body irradiation (TBI) or in the number of viral infections post-HSCT (*p* = 0.29) between patient with NIPS and controls. GvHD prophylaxis was cyclosporine-based for majority of the patients in both groups. Patients with NIPS had more often both acute grade 2–4 GvHD (*p* = 0.004) as well as chronic GvHD (*p* = 0.0004) as compared to the controls. Demographic data of 12 patients with NIPS and their controls are presented in Table 1.

### Ventilatory function

The detailed information about polysomnography measurements is presented in Table 2. The median number of polysomnographies performed for the 12 symptomatic patients was 1 (IQR 1–4, range 1–13). Children with nighttime non-invasive ventilation support (Children #1–#4) had polysomnography analysis also during ventilation support (Fig. 1).

**Table 2:** Details of polysomnography studies.

Number of studied patients	12
Sleep characteristics	
Total sleep time (min)	442 (400–481)
Non-REM (min)	351 (310–369)
REM (min)	103 (66–123)
Sleep efficiency (%)	91 (81–92)
Breathing characteristics	
Central apnea index	0,0 (0–0.2)
Obstructive apnea/hypopnea index	0.1 (0–0.7)
EtCO_2_ maximum	6.3 (5.8–6.8)
SpO_2_ mean	95 (93–96)
Work of breathing (0–2/normal–laborious)	1.0 (0–2.0)

*REM* rapid eye movement sleep, *EtCO*_*2*_ end-tidal carbon dioxide, *SpO*_*2*_ pulse oximeter oxyhemoglobin saturation.

**Fig. 3:**
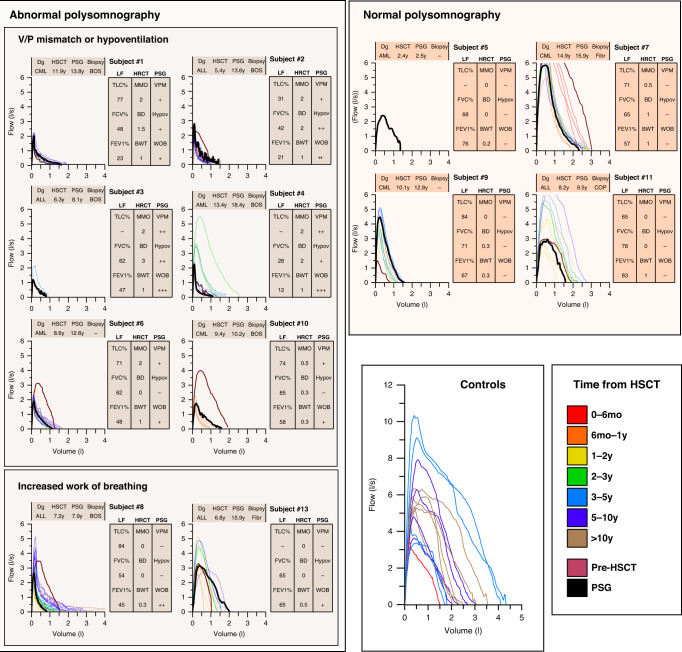
Individual spirometry, diffusion capacity, lung imaging, and polysomnography test results. Children are grouped according to polysomnography results. Children numbered 1, 2, 3, 4, 6, 8, and 10 had spirometry results indicating small airway obstruction. These children presented with a ventilation-to-perfusion mismatch in PSG. In addition, patients numbered 1, 2, 3, and 4 had REM sleep-related hypoventilation. Children numbered 8 and 13 had increased work of breathing but no hypoventilation is PSG. Other children (5, 7, 9, 11) had restrictive lung function in spirometry, no ventilation-to-perfusion-mismatch, no hypoventilation nor increased work of breath in PSG. Controls presented with normal spirometry. In spirometry figures, the spirometry test curve with shortest time window from polysomnography is indicated by heavier black line. BOS bronchiolitis obliterans syndrome, Dg diagnosis, HRCT high-resolution computer tomography, HSCT allogeneic hematopoietic stem cell transplantation, LF lung function testing, PSG polysomnography, BWT bronchial wall thickening, WOB work of breathing, V/P ventilation to perfusion, VPM ventilation-to-perfusion mismatch, MMO mosaic pattern, BD bronchial dilation.

**Fig. 4:**
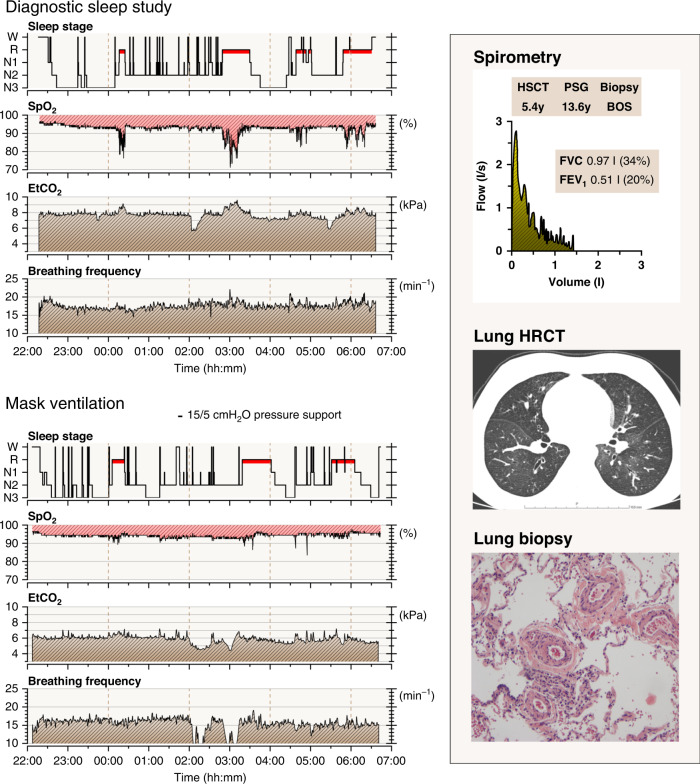
An example of polysomnography recording summary, spirometry, lung high-resolution computer tomography, and biopsy finding in a child (#2) with obstructive lung function testing and sleep-related hypoventilation. Polysomnography (PSG) summary is presented with two consecutive nights: a diagnostic sleep study followed by a study with a non-invasive mask ventilation support in spontaneous mode with inspiratory pressure of 15 cmH_2_O and expiratory pressure of 5 cmH_2_O. The child presented with exercise dyspnea, spirometry indication severe small airway obstruction, mosaic pattern in lung imaging, obliterative bronchiolitis in lung biopsy (BOS), and hypoventilation during sleep. REM-sleep periods were related with severe SpO_2_ desaturations. EtCO_2_ end-tidal carbon dioxide, HSCT allogeneic hematopoietic stem cell transplantation, SpO_2_ pulse oximeter oxyhemoglobin saturation.

Chronic lung problem behaved in a logical manner (Fig. 3): children who developed clearly obstructive lung function (*n* = 7) presented with mosaic pattern in lung HRCT (6/7), had BOS in lung biopsy (6/6), and they developed sleep-related breathing disorder which was related to the degree of decrease in spirometry values. These children showed increased work of breathing during sleep (7/7), some degree of ventilation-to-perfusion mismatch (VPM; 6/7), and most severely decreased lung function was related with hypoventilation during sleep (*n* = 4, children #1, #2, #3, #4), especially in REM-sleep (Figs. 3 and 4). Only one (1/5, #13) of the children who had more restrictive pattern in spirometry (children #5, #7, #9, #11, and #13), showed increased work of breathing but none presented with decreased oxygenation or hypoventilation (Fig. 3). None of the children with NIPS showed upper airway related or central breathing problems, such as obstructive sleep apnea, partial upper airway obstruction with airflow limitation, central sleep apnea, or central hypoventilation.

VPM in polysomnography recording was found in 5 children (50%). Median FEV_1_ value at the time of ventilation-perfusion mismatch was 45% of predicted normal median (IQR 23–51%). Hypoventilation during REM sleep was seen in four of these six children (67%). Median FEV_1_ value at the time of hypoventilation was 29% (IQR 15–44%). For comparison, the median FEV_1_ value 2–5 years post- HSCT in the control group was 89% (IQR 79–95%). The difference between children with NIPS and the controls is clear, but because of small sample size no statistical evaluation can be done.

The median time from HSCT to the development of lung symptoms and decreased pulmonary function was 7.5 (IQR 6–9) months.

Diffusion capacity study was performed to the children with NIPS. Seven children had residual volume over 120% measured at least once during the follow-up. Median TLC was 83% (IQR 63–85%), RV 141% (IQR 117–153%), and DLCOc/VA 1.89 mmol/min/kPa/l (IQR 1.47–2.09 mmol/min/kPa/l).

The individual lung HRCT and lung biopsy findings are presented in Figs. 3 and 4 in combination with polysomnography findings. In short, mosaic pattern was observed in 7/12 HRCT images. Lung biopsy showed BOS in 6/12 patients with NIPS.

### The NIH criteria

Five out of 12 children with NIPS fulfilled the modified NIH criteria of BOS (#1, #2, #4, #6 and #7).^9^ The reasons for not fulfilling the criteria were missing residual volume (RV) measurement (*n* = 3), RV under 120% (*n* = 2), and no air trapping in HRCT (*n* = 5). Only four cases met the original NIH criteria by Filipovich et al. (#1, #2, #4 and #10).^16^ Most of our children with NIPS did not meet the NIH criteria of FEV_1_/FVC ratio <0.7.^16^

## Discussion

We show that pediatric patients with severe NIPS and obstructive lung function have a significant risk for sleep-related breathing disorder with VPM, increased work of breathing, and REM-sleep-related hypoventilation. In contrast, patients in this cohort with restrictive lung function had normal polysomnography.

The patients with BOS developed sleep-related breathing disorder, in addition to early obstructive lung function and mosaic pattern in the lung HRCT. These children had increased work of breathing during sleep, modestly decreased baseline oxygenation, and VPM while severely affected children presented with hypoventilation during sleep with REM sleep predominance. In addition, we show that children with chronic respiratory symptoms after allogeneic HSCT have a clear decrease in lung function testing early after HSCT. This is supported by previous studies.^4–6,17^

In our study population, only 4 children (33%) fulfilled the original NIH criteria and 5 (42%) the modified NIH criteria.^9,16^ Most of our patients did not meet the criteria of FEV_1_/FVC < 0.7 and those who did, developed it later in the course of the disease. In NIH criteria for BOS, lung HRCT findings are ranked high.^16^ Children with severely decreased pulmonary function may not always be able to exhale properly to make air trapping or mosaic pattern visible in lung HRCT. Our results support previous findings that spirometry showing a persistent obstructive lung function is a better marker of BOS than HRCT.^4,6,8,9^ Jagasia and associates published new diagnostic criteria for BOS in 2015 to enhance sensitivity.^18^ Despite these changes, the NIH criteria may not be sensitive enough to detect BOS early.

The incidence of BOS in adult population is estimated at 5–10% of allogeneic HSCT recipients, and 16% of patients with cGvHD.^9^ The mortality rate in adult patients is reported between 13 and 100%.^11,19^ Only few small pediatric cohorts (*n* = 10–40) have been reported showing the incidence of BOS at 2–8%.^11,20,21^ The mortality rates in pediatric patients has been between 11 and 67%, congruently based on small cohorts.^11,20^ In our study population, the mortality rate in patients with BOS was 50%.

BOS is usually diagnosed at the stage when airways are already severely affected. Known risk factors for developing BOS is another chronic GvHD manifestation and patients with a history of lung disease.^6,16,19,22,23^ In our series of children with NIPS, only one did not have other manifestation of cGvHD (#5). In addition, risk factor for NIPS was acute GvHD grade 2–4. This is in line with the literature.^4,8,11^ Pretransplant lung function was normal in all but one with NIPS and one in control group, thus its role as a risk factor for BOS could not be evaluated as in previous studies.^4,6,20,22^

Our findings support the use of polysomnography as a part of lung function testing in children with BOS and severely obstructive lung function. In our population, nighttime ventilation support improved daytime physical performance and thus supposedly improved the quality of life. However, we do not expect that ventilation support would have significantly affected long-term survival.

The small number of patients with polysomnography and lack of polysomnography in controls may cause bias in our results. However, we carefully and critically analyzed the versatile data of patients with NIPS and believe that the patients diagnosed with BOS could reliably be identified. We did not uniformly have lung function tests available from 6 weeks or 3 months timepoints after HSCT, which could have strengthened the predictive value of our data.

In conclusion, we show that patients with BOS may develop sleep-related breathing disorder, and may benefit from polysomnography follow-up. Children with BOS showed increased work of breathing during sleep, had some degree of VPM, and hypoventilation during REM sleep. In contrast, children with NIPS but no BOS, had restrictive lung function test results, and little or no risk for VPM or sleep-related hypoventilation.

## Data Availability

The datasets generated during and/or analyzed during the current study are available from the corresponding author on reasonable request.
